# Regular Meeting of the Chicago Medical Society

**Published:** 1872-01-01

**Authors:** 


### Society Reports.

### REGULAR MEETING OF THE CHICAGO MEDICAL SOCIETY.

Office of Dr J. P. Ross, Dec. 4th, 1872.

Dr. R. G. Bogue in the chair.

Dr. Stillians exhibited a mature fœtus, of which the following is the history: Two days previously a request was left at the Doctor's office to call sometime during the day, and in the evening when he arrived at the bedside he found the fœtus expelled and a midwife trying to remove the placenta. The attendant directed the Doctor's attention to the fœtus, at the same time affirming that the head presented, and that no force had been used in its extraction. The scalp was found to be lacerated, the neck presenting a gaping, lacerated wound which extended from one angle of the jaw to the other, the cranial cartilage comminuted, and the brain to be still within the vagina. The body of the fœtus was hard, shining, swollen and œdematous, the upper extremities imperfectly developed, the lower malformed, having the appearance of an extra joint between the knee and ankle, and each presenting a fracture just below this false joint.

It was the opinion of the members present that the presentation had been pelvic, and that powerful traction had been made on the lower extremities.

Dr. Quine related the case of a stout, muscular blacksmith aged 49, who until May, 1869, had enjoyed uninterrupted health, but at that time having slept with insufficient covering in a wood-shed, was attacked by a severe chill, followed by protracted fever, distressing cough, copious rusty expectoration, profuse diarrhœa and rapid emaciation. The parents of the patient were living and in the enjoyment of good health, and no members of the family had died of pulmonary disease. On examination the patient was found considerably emaciated, depression above and below the clavicles, elevation of shoulders, diminished expansion of both sides, depression of intercostal spaces during inspiration, a dull sound on percussing the upper two-thirds of left side and upper third of right, but a tympanitic sound on percussing the right mammary and right infraspinous regions. Auscultation; crackling inspiration, prolonged expiration, and increased vocal resonance over the upper two-thirds of the left side and upper third of the right, over right infraspinous region gurgling with respiratory movements and on speaking the distinct transmission of articulated words. The last phenomenon was also discovered in the left apex anteriorly. The patient failed rapidly and died on the 8th day of October. The treatment consisted of cod-liver oil ℨ is. three times daily, iodide and bromide of potassium each gr. x. four times a day, anodynes as required, and bitter tonics.

*Post Mortem.*—Chest only examined. On removing the sternum and cartilages, about a pint of pus escaped from the right side. The right pleural cavity was obliterated by adhesion of the parietal and visceral pleura. A large cavity was found in the lower third of the right lung, bounded in front by visceral pleura, behind, above and below by a thin stratum of lung tissue, and a small cavity in the apex of the left lung, bounded on all sides by lung tissue. Both lungs were infiltrated with granular caseous matter.

The Vienna Medical Faculty is composed of 135 "kollegian," 19 ordinary, and 20 extraordinary professors, and eight privat-docenten.

